# Agreement of Anterior Segment Parameter Measurements With CASIA 2 and IOLMaster 700

**DOI:** 10.3389/fmed.2022.777443

**Published:** 2022-02-11

**Authors:** Xiaoting Ruan, GuangYao Yang, Zhaoxia Xia, Jiaqing Zhang, Xiaoxun Gu, Yuan Tan, Zhenzhen Liu, Lixia Luo

**Affiliations:** ^1^State Key Laboratory of Ophthalmology, Zhongshan Ophthalmic Center, Sun Yat-sen University, Guangdong Provincial Key Laboratory of Ophthalmology and Visual Science, Guangdong Provincial Clinical Research Center for Ocular Diseases, Guangzhou, China; ^2^Department of Ophthalmology, The Sixth Affiliated Hospital of Sun Yat-sen University, Guangzhou, China

**Keywords:** CASIA 2, IOLMaster 700, anterior segment optical coherence tomography, agreement, anterior segment parameters

## Abstract

**Purpose:**

To compare the difference and agreement in central corneal thickness (CCT), keratometry (K), anterior chamber depth (ACD), aqueous depth (AQD), and lens thickness (LT) measured with CASIA 2 and IOLMaster 700 in patients with cataract.

**Methods:**

A total of 81 patients with cataract (81 eyes) scheduled for phacoemulsification were prospectively collected from March to May, 2020 in the cataract department of Zhongshan Ophthalmic Center, Sun Yat-sen University, including 43 males and 38 females with age of 61.5 ± 10.6 years. CCT, anterior K_f_, anterior K_s_, real K_f_, real K_s_, ACD, AQD, and LT were measured with CASIA 2 and IOLMaster 700. Paired *t*-test, intraclass correlation coefficients (ICCs), 95% limit of agreement (95% LoA), and Bland-Altman plots were performed and used to analyze the difference and agreement between the two devices.

**Results:**

There was no statistically significant difference in anterior K measurement with the CASIA 2 (44.3 ± 1.66 mm) and IOLMaster 700 (44.31 ± 1.67 mm, *P* = 0.483). Differences among the CCT, anterior K_f_, real K_f_, real K_s_, ACD, AQD, and LT measured by the two instruments were statistically significant (*P* < 0.001). The ICCs of CCT, anterior K_f_, anterior K_s_, real K_f_, real K_s_, ACD, AQD, and LT measurements between the two devices were 0.892, 0.991, 0.991, 0.827, 0.817, 0.937, 0.926, and 0.997, respectively. The 95% LoA between CASIA 2 and IOLMaster 700 was −30.06 to 0.43 μm for CCT, −0.3 to 0.48 D for anterior Kf, −0.46 to −0.43 D for anterior Ks, −1.49 to −0.49 D for real Kf, −1.62 to −0.49 D for Real Ks, −0.03 to 0.24 mm for ACD, 0.04 to 0.25 mm for AQD, and −0.06 to 0.09 mm for LT.

**Conclusion:**

Anterior K_f_, anterior K_s_, ACD, AQD, and LT have excellent agreement between the two devices. CCT, real K_f_, and real K_s_ have moderate agreement between the two devices. It is recommended to use anterior K_f_, anterior Ks, ACD, AQD, and LT interchangeably between CASIA 2 and IOLMaster 700.

## Introduction

At present, modern cataract surgery has shifted from restoring vision to refractive surgery. Surgeons need to customize the refractive prediction of different patient to meet their visual expectations. Therefore, the accuracy of intraocular lens (IOL) calculation formula is very important. Olsen T pointed out that keratometry (K) measurement error accounted for 22% of total prediction error, and that ACD measurement error accounted for 42% (1, 2). Therefore, accurate preoperative ocular biometry is very important for patients with cataract to obtain good refractive status.

At present, many methods of measuring anterior segment parameters are used in clinical practice; however, the same parameter measured by different instruments often has systematic deviation. Previous studies have compared the agreement of IOLMaster 700 with IOLMaster 500 (3), Lenstar LS 900 (4), Pentacam AXL (5), OA-2000 (6), Pentacam HR, vs. cirrus HD-OCT (7) in the measurement of ACD, central corneal thickness (CCT) and keratometry. The results show that IOLMaster 700 has good agreement with other instruments. For example, ACD AL, K1, and K2 can be used interchangeably between IOLMaster 700 and IOLMaster 500 in clinical practice. IOL Master has now become the gold standard for clinical measurement of ocular parameters (8–10), and is widely used in preoperative evaluation of patients with cataract.

CASIA 2 (Tomey Corporation, Nagoya, Japan), the second generation of anterior segment OCT has emerged in recent years, combines Fourier domain technology and frequency sweep source OCT technology to further optimize scanning speed (50,000 A-scans/s), scanning depth (16 × 16 × 13 mm), scanning density, and imaging resolution. However, its measurement accuracy remains unclear (11–15). As we all know, if a new device wants to be recognized by clinicians and be widely used in clinical practice, it will be compared with the currently recognized instrument. As far as we know, there are currently few studies comparing CASIA 2 with IOLMaster 700 in anterior segment parameter measurements (16, 17). The sample size of our study (81 eyes of 81 patients) is larger than the previous study comparing CASIA2 and IOLMaster 700 (48 eyes of 48 patients and 47 eyes of 29 patients, respectively).

In this study, we aimed to compare the difference and agreement between CASIA 2 and IOLMaster 700 in anterior segment parameter measurements.

## Methods

### Subjects and Settings

Eighty one patients with cataract (81 right eyes) scheduled for phacoemulsification at Zhongshan Ophthalmic Center of Sun Yat-sen University from March 2020 to May 2020 were enrolled in this study, including 43 males and 38 females, aged 61.5 ± 10.6 years. Exclusion criteria were as follows: patients with dry eye, keratitis, pterygium, corneal scar, keratoconus and other ocular surface diseases; glaucoma, uveitis, retinal detachment, and other intraocular diseases, nystagmus leading to failure of fixed vision, and those who had been wearing contact lenses for a long time, and those who had a history of ocular trauma and eye surgery were excluded. This study was in line with the declaration of Helsinki and was approved by the ethics committee of Zhongshan Ophthalmic Center. All the patients signed informed consent.

### Anterior Segment Scanning

Anterior segment measurements with CASIA2 and IOLMaster 700 were performed in a same dark room by two examiners. After 30 min of mydriasis with 1% tropicamide, the patients were asked to sit in front of the equipment in the dark room. After a complete blink, the patients should focus on the cursor in the instrument and open their eyes as much as possible to complete a measurement. For CASIA2, the examiners evaluated image quality during inspection and selected the results with acceptable quality for statistical analysis. For IOL Master700, images with high quality evaluated by built-in software were enrolled for analysis. All the parameters involved in this study measured with CASIA2 and IOLMaster 700 were automatically measured with the built-in software. Each participant has one measurement by CASIA2 and IOLMaster 700. The parameters we analyzed in this study include CCT, anterior K_f_, anterior K_s_, real K_f_, real K_s_, ACD, AQD, and LT.

### Data Analysis

The SPSS statistical software (SPSS Statistics version 22.0; IBM Corp., Armonk, NY, United States) was used to analyze the right eye data of all the patients. All continuous variables were expressed using mean ± standard deviation. Paired *t*-test was performed to analyze the difference of measurement data that conform to normal distribution, and rank sum test is performed for measurement data that do not conform to normal distribution. Intraclass correlation coefficients (ICCs) and Bland-Altman plots were used to assess the agreement of the two devices. ICCs are a widely used reliability index in reliability and agreement analyses. This index ranges between 0 and 1, with values closer to 1 representing stronger reliability or agreement (18). ICCs were estimated and calculated using SPSS statistical package version 22 (SPSS Inc, Chicago, IL, United States) based on a single measurement, absolute-agreement, two-way random-effects model. Statistical significance was defined as *P* < 0.05.

## Results

A total of 81 patients (81 right eyes) with an average age of 61.5 ± 10.6 years were included in this study. Table 1 summarizes the baseline profile of the patients.

**Table 1:** Baseline characteristics of the participants.

**Characteristics**	**Cataract**
	**(*N* = 81 persons, 81 eyes)**
**Age** (Mean ± SD, years)	61.5 ± 10.6
**Gender**	
Male, *n* (%)	43 (53.1)
Female, *n* (%)	38 (46.9)

*SD, Standard deviation*.

The measurement results with CASIA 2 and IOLMaster 700 are shown in Table 2. The normal distribution of all the measured parameters was analyzed, and W test showed that all the parameters were in accordance with normal distribution. The data were analyzed by paired *t*-test. The average difference in anterior K_f_ was 0.09 ± 0.2 D; that in anterior K_s_ was −0.02 ± 0.23 D; that in real K_f_ was −0.99 ± 0.25 D; that in real K_s_ was −1.06 ± 0.29 D; that in CCT was −14.81 ± 7.78 mm; that in ACD was 0.13 ± 0.05 mm; and that in AQD was 0.15± 0.05 mm. Except for anterior K_s_ (*P* = 0.483 >0.05), differences in the other parameters were statistically significant (*P* < 0.001).

**Table 2:** Comparison of anterior segment parameter measurements with CASIA 2 and IOLMaster 700.

**Parameters**	**CASIA2**	**IOLMaster 700**	**Mean diff ±SD**	***P*-value**	**ICC, *P*-value**
CCT (μm)					
Mean ± SD	530.96 ± 34.25	545.78 ± 34.40	−14.81 ± 7.78	*P* < 0.001	0.892, *P* < 0.001
Range	452–644	458–640			
Anterior K_f_ (D)					
Mean ± SD	43.53 ± 1.59	43.45 ± 1.61	0.09 ± 0.20	*P* < 0.001	0.991, *P* < 0.001
Range	39.90–47.10	39.85–47.00			
Anterior K_s_ (D)					
Mean ± SD	44.30 ± 1.66	44.31 ± 1.67	−0.02 ± 0.23	*P* = 0.483	0.991, *P* < 0.001
Range	40.10–47.70	40.09–47.98			
Real K_f_ (D)					
Mean ± SD	42.46 ± 1.57	43.45 ± 1.61	−0.99 ± 0.25	*P* < 0.001	0.827, *P* < 0.001
Range	38.90–46.10	39.85–47.00			
Real K_s_ (D)					
Mean ± SD	43.26 ± 1.62	44.31 ± 1.67	−1.06 ± 0.29	*P* < 0.001	0.817, *P* < 0.001
Range	39.30–46.60	40.09–47.89			
ACD (mm)					
Mean ± SD	3.36 ± 0.4	3.23 ± 0.39	0.13 ± 0.05	*P* < 0.001	0.937, *P* < 0.001
Range	2.18–4.13	2.07–4.2			
AQD (mm)					
Mean ± SD	2.83 ± 0.4	2.68 ± 0.39	0.15 ± 0.05	*P* < 0.001	0.926, *P* < 0.001
Range	1.65–3.78	1.52–3.66			
LT (mm)					
Mean ± SD	4.38 ± 0.54	4.36 ± 0.52	0.02 ± 0.04	*P* < 0.001	0.997, *P* < 0.001
Range	3.23–5.67	3.24–5.67			

*K, keratometry; ACD, anterior chamber depth; AQD, aqueous depth; LT, lens thickness; CCT, central corneal thickness; D, diopter; mm, millimeter; μm, micron; SD, standard deviation; diff, difference; ICC, intraclass coefficient*.

The ICC of CCT, anterior K_f_, anterior K_s_, real K_f_, real K_s_, ACD, AQD, and LT were 0.892, 0.991, 0.991, 0.827, 0.817, 0.937, 0.926, and 0.997, respectively. The ICC showed that CASIA 2 had a good agreement with IOLMaster 700 in anterior K_f_, anterior K_s_, ACD, AQD, and LT. Also, there was a moderate agreement between the two devices in the measurement of CCT, real K_f_, and real K_s_.

**Figure 1:**
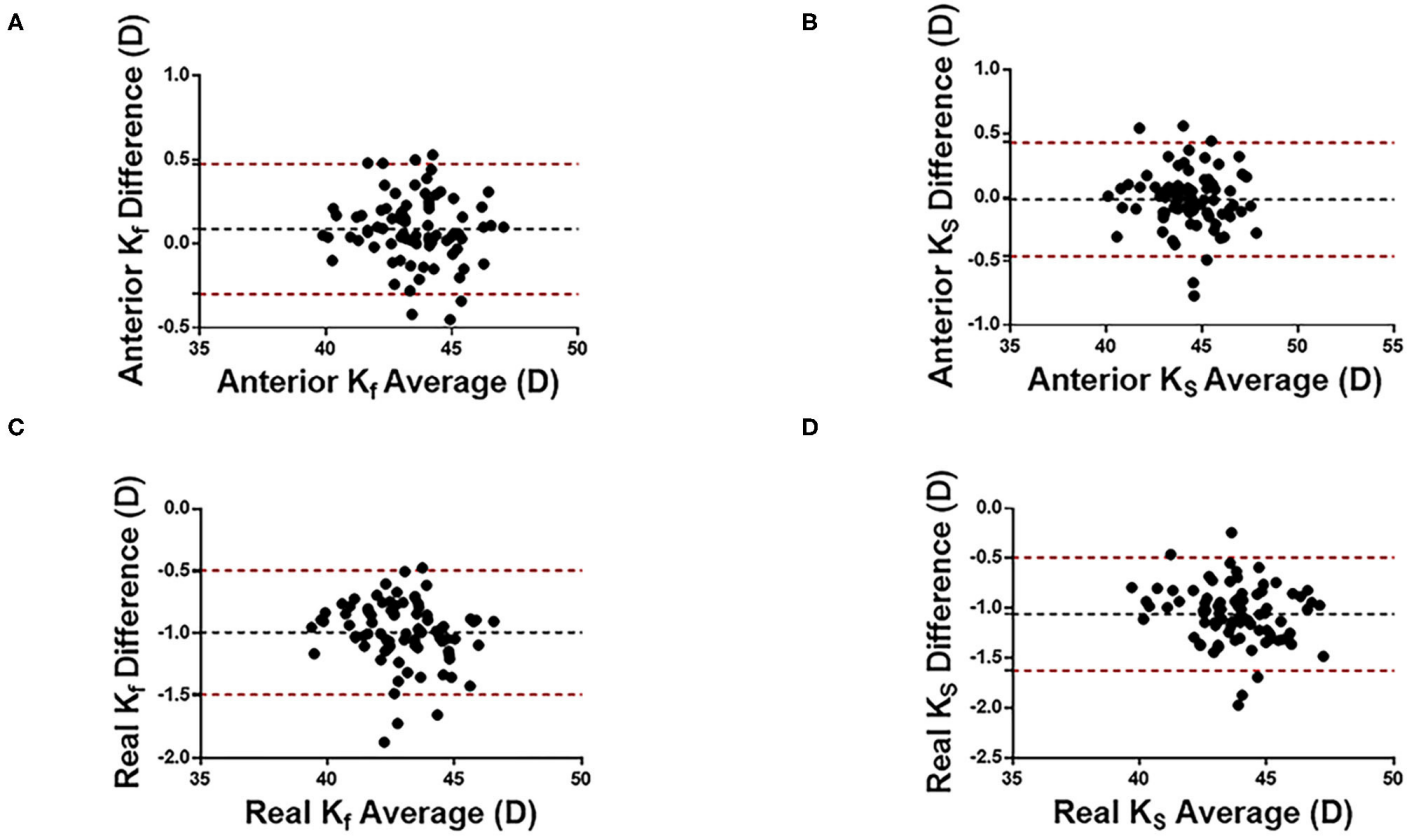
Bland-Altman plots of cornea curvature measurement with CASIA2 and IOL-mater700. Kf, flat keratometric power; Ks, steep keratometric power. **(A)** Anterior Kf, **(B)** Anterior Ks, **(C)** Real Kf, and **(D)** Real Ks. Black dotted lines indicate the bias between both devices and red dotted lines indicate the 95% confidence interval for the difference.

**Figure 2:**
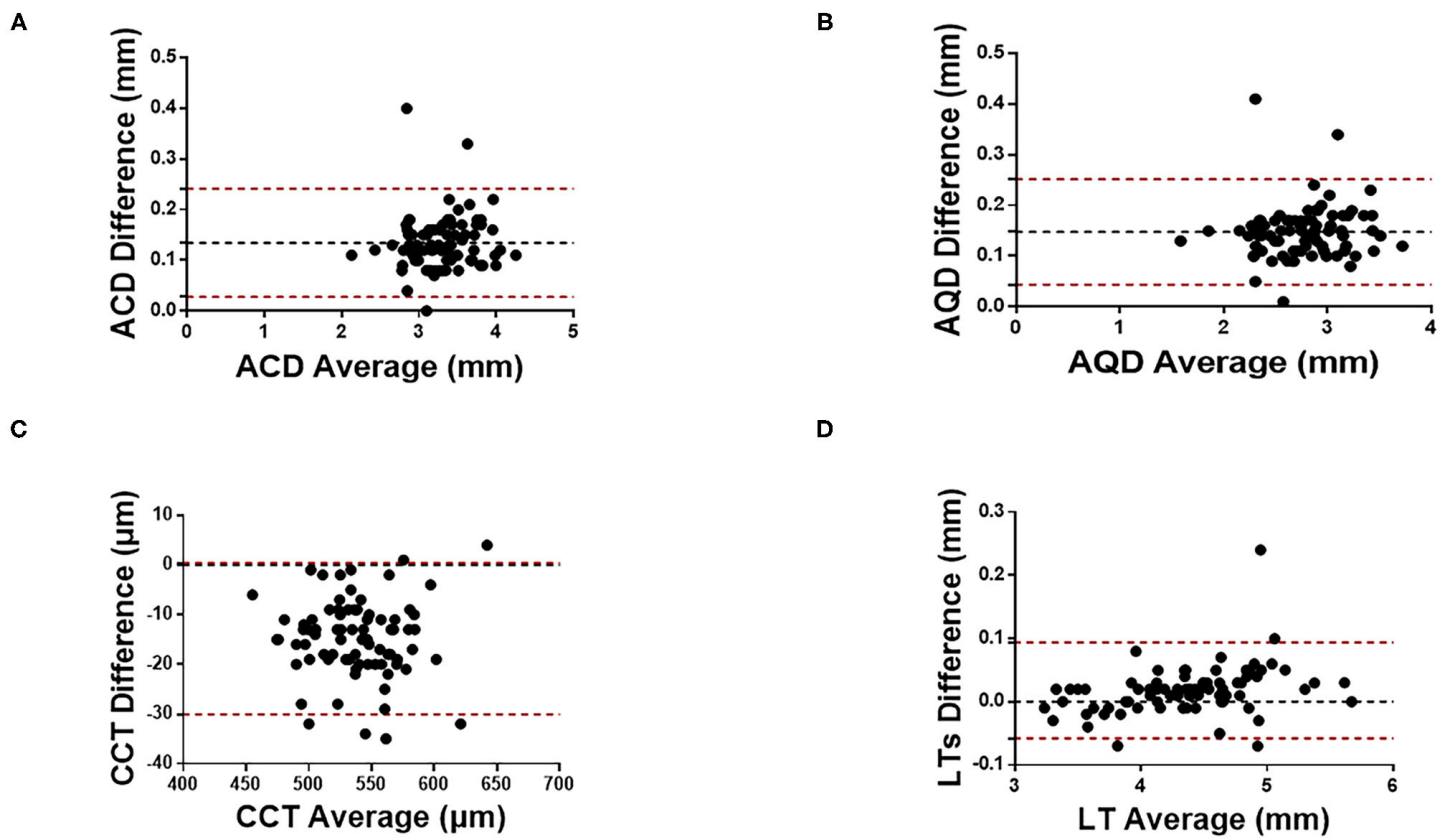
Bland-Altman plots of **(A)** ACD, **(B)** AQD, **(C)** CCT, and **(D)** LT measurements with CASIA2 and IOL-Master 700. ACD, anterior chamber depth; AQD, aqueous depth; CCT, central cornea thickness; LT, lens thickness.

Figures 1, 2 show the Bland Altman plot for each parameter. This study found that the anterior K_f_, anterior K_s_, ACD, AQD, and LT measurements between CASIA 2 and IOLMaster 700 had a narrow 95% LoA of 0.3 to 0.48 D, −0.46 to 0.43 D, 0.03 to 0.24 mm, 0.04 to 0.25 mm, and −0.06 to 0.09 mm, respectively, indicating a good agreement in those parameters. The CCT, real K_f_, and real K_s_ measurements between the two instruments had a little bit broad 95% LoA of −30.06 to 0.43 μm, −1.49 to −0.49 D, and −1.62 to −0.49 D, respectively, indicating a moderate agreement in those parameters.

## Discussion

This study analyzed the agreement between CASIA 2 and IOLMaster 700 in anterior K_f_, anterior K_s_, real K_f_, real K_s_, CCT, ACD, AQD, and LT. The comprehensive analysis results showed that there was a good agreement in the anterior K_f_, anterior K_s_, ACD, AQD, and LT measurements and a moderate agreement in the CCT, real K_f_ and real K_s_ measurements.

The agreement between the two instruments was high in terms of anterior K_f_ and anterior K_S_. The ICC results showed that there were high agreements in the measurements of anterior K_f_ (ICC:0.991, 95% LoA: −0.30 to 0.48 D), anterior K_s_ (ICC:0.991, 95% LoA: −0.46 to 0.43 D), ACD (ICC:0.937, 95% LoA:0.03 to 0.24 mm), AQD (ICC: 0.926, 95% LoA: 0.04 to 0.25 mm), and LT (ICC:0.997, 95% LoA: −0.06 to 0.09 mm), and moderate agreements in the measurements of CCT (ICC =0.892, 95% LoA: −30.06 to 0.43 μm), real K_f_ (ICC:0.827, 95% LoA: −1.49 to −0.49 D), and real K_s_ (ICC: 0.817, 95% LoA: −1.62 to −0.49 D).

In daily practice, the difference of corneal curvature within 0.5D is clinically acceptable. Among the four kind of corneal curvature parameters, the measurements of anterior K_f_ and anterior K_s_ were close between CASIA2 and IOLMaster700, while results of real K_f_ and real K_s_ showed significant difference between two machines. So anterior K_f_ and anterior K_s_ would be used interchangeably with CASIA2 and IOL Master700. In a previous study, Wylegala et al. compared CCT and anterior corneal curvature measured with CASIA 2, Galilei G6 (Scheimpflug analyzer), and RevoNX (spectral domain OCT) (14). Their results showed good agreement in the measurements of anterior K_f_ and anterior K_s_ and moderate agreement in CCT measurements between CASIA2 and Galilei.

Our results show that the difference in real K_f_ and real K_S_ measured with CASIA 2 and IOLMaster 700 is may be due to the different principles of the two instruments. CASIA 2 takes 32 measuring points from the central 3-mm area of the cornea and connects the two points symmetrically centered on the corneal apex to form 16 straight lines. Among these 16 lines, the line with strongest diopter is K_s_, and the radius of curvature in the direction with an angle of 90° with K_s_ is K_f_. Using this principle, CASIA 2 can calculate the anterior and posterior corneal curvatures and the real corneal curvature by paraxial calculation using the Gullstrand model eye refractive index. The IOLMaster 700, based on frequency sweep technology, can only measure the corneal curvature of the anterior surface, and its measurement principle is to obtain eight measuring points from 2.5 mm in the center of the cornea. According to projection images of the eight measuring points, an ellipse is fitted. The short radius of the ellipse is K_2_, and the radius in the direction of 90° with K_2_ is considered as K_1_. Therefore, the difference in measurement principle may be the main reason for the difference in real K_f_ and real K_S_ measurement results between CASIA 2 and IOLMaster 700. As suggested by previous studies, our results show that the parameters real K_f_ and real K_S_ of the two instruments should not be used interchangeably.

The agreement in ACD, AQD, and LT measured with CASIA 2 and IOLMaster 700 was high and CCT measurement had a moderate agreement. CASIA 2 has a different agreement with CCT, ACD, AQD, and LT measured by other instruments. Previous study has shown a high correlation of measurements result of ACD, anterior chamber width and other parameter between CASIA2 and time-domain AS-OCT (15), however, there is a constant proportion of deviation in most parameters, so it is not recommended to use the parameters interchangeably. Li et al. evaluated the agreement of CASIA2 and Pentacam, and their results showed good agreement in CCT and ACD measurements (19). Fukuda et al. showed that there was no statistically significant difference in CCT, ACD, AQD, and LT measured with CASIA 2 and CASIA 1 (13).

CASIA 2 is also consistent with IOLMaster 700 in CCT, ACD, AQD, and LT, even if there are acceptable differences. The differences between both instruments in the measurement of CCT, ACD, AQD, and LT were possible due to different technology and different image analysis principles. CASIA 2 utilizes a wavelength of 1,310 nm, and its axial resolution is <10 μm (20), while IOLMaster 700 uses a tunable laser with an average wavelength of 1,055 nm and an axial resolution of 22 μm (4). Therefore, CASIA 2 can penetrate tissues better with longer wavelength and identify the boundary of a single structure with better accuracy. Second, CASIA 2 uses 1,310-nm infrared light and high-speed linear scanning (50,000A-scans/s), and the whole measurement time is 0.3 s, which can minimize the influence of measurement light on pupil movement and shrinkage (21). However, the scanning speed of IOLMaster 700 is 2,000 A-scans/s, so the examination process needs to last 3.5 s (22). The difference between the two may lead to a different coordination degree of subjects. The shortened scanning time can reduce the influence of motion artifacts caused by involuntary eye movement and patient pressure, making it easier for patients to tolerate and cooperate with the examination, thus affecting the agreement of the results of the two instruments (23). Finally, IOLMaster 700 is based on the swept optical biological technology, and CASIA 2 is the second generation of OCT that integrates swept OCT technology and Fourier domain technology. Many studies have found that time domain OCT, Fourier domain OCT, and swept OCT devices have differences in measurement of corneal thickness, nerve fiber layer thickness, and macular thickness (24–27); that is, different OCT measurement principles will also lead to differences in measured values.

In clinical application, CASIA2 is rather an OCT, which is mainly used to measure biological parameters of an ocular anterior segment, such as calculating the size of an ICL. IOLMaster 700 is a biometer, which is mainly used to measure axial length and calculate IOL power. The anterior and posterior of the cornea are crucial to patients who will have an implant of a trifocal lens. The posterior corneal surface curvature with CASIA2 is measured by simulation. The type of IOL Master 700 we used in this study cannot measure posterior corneal surface curvature. The new type of IOL Master, 700 TK, can measure posterior corneal surface curvature directly. Currently, the golden standard in cornea measurement is Pentacam. There are few studies on agreement between CASIA2 and Pentacam that showed the agreement in anterior and posterior corneal curvature is acceptable (28). There is currently no study on agreement of IOLMaster 700 TK and Pentacam in posterior corneal surface curvature.

There are several limitations in this study. In this study, we included only eyes without a history of underlying eye diseases and eye surgery. We believe that the agreement of measurement results is likely to be lower for patients with underlying eye diseases (such as abnormal corneal shape and keratoconus.). Second, only patients with cataract were included in this study. More multicenter clinical trials are needed to verify the validity and reliability of CASIA 2 in different populations. At the same time, both CASIA 2 and IOLMaster 700 are optical imaging OCTs, which will cause geometric optical distortion in the imaging process. The correction methods of different manufacturers may be inconsistent. However, due to the protection of patent law, we cannot know the specific correction scheme.

## Conclusion

Our study compared CASIA 2 with IOLMaster 700. It is recommended to use anterior K_f_, anterior K_s_, ACD, AQD, and LT between the instruments interchangeably. In addition, CASIA 2, with high penetrability and high resolution, is more valuable for the diagnosis and treatment of ophthalmic diseases.

## Data Availability Statement

The raw data supporting the conclusions of this article will be made available by the authors, without undue reservation.

## Ethics Statement

The studies involving human participants were reviewed and approved by the Ethics Committee of Zhongshan Ophthalmic Center. The patients/participants provided their written informed consent to participate in this study. Written informed consent was obtained from the individual(s) for the publication of any potentially identifiable images or data included in this article.

## Author Contributions

ZL and LL conceived and designed the research. GY, XR, XG, and JZ collected the data. ZX, GY, and XR analyzed the data. GY and XR wrote the manuscript. LL and ZX critically revised the manuscript. All authors discussed the results and provided comments on the manuscript. All authors contributed to the article and approved the submitted version.

## Funding

This study was supported by the National Natural Science Foundation of China (81873675 and 81770905) and the Construction Project of High-Level Hospitals in Guangdong Province (303020102).

## Conflict of Interest

The authors declare that the research was conducted in the absence of any commercial or financial relationships that could be construed as a potential conflict of interest.

## Publisher's Note

All claims expressed in this article are solely those of the authors and do not necessarily represent those of their affiliated organizations, or those of the publisher, the editors and the reviewers. Any product that may be evaluated in this article, or claim that may be made by its manufacturer, is not guaranteed or endorsed by the publisher.
